# Detection of Intracellular Factor VIII Protein in Peripheral Blood Mononuclear Cells by Flow Cytometry

**DOI:** 10.1155/2013/793502

**Published:** 2013-02-28

**Authors:** Gouri Shankar Pandey, Sandra C. Tseng, Tom E. Howard, Zuben E. Sauna

**Affiliations:** ^1^Laboratory of Hemostasis, Division of Hematology, Center for Biologics Evaluation and Research, Food and Drug Administration, 29 Lincoln Drive, Bethesda, MD 20892, USA; ^2^Department of Pathology and Laboratory Medicine, Veterans Affairs Greater Los Angeles Healthcare System, Los Angeles, CA 90073, USA

## Abstract

Flow cytometry is widely used in cancer research for diagnosis, detection of minimal residual disease, as well as immune monitoring and profiling following immunotherapy. Detection of specific host proteins for diagnosis predominantly uses quantitative PCR and western blotting assays. In this study, we optimized a flow cytometry-based detection assay for Factor VIII protein in peripheral blood mononuclear cells (PBMCs). An indirect intracellular staining (ICS) method was standardized using monoclonal antibodies to different domains of human Factor VIII protein. The FVIII protein expression level was estimated by calculating the mean and median fluorescence intensities (MFI) values for each monoclonal antibody. ICS staining of transiently transfected cell lines supported the method's specificity. Intracellular FVIII protein expression was also detected by the monoclonal antibodies used in the study in PBMCs of five blood donors. In summary, our data suggest that intracellular FVIII detection in PBMCs of hemophilia A patients can be a rapid and reliable method to detect intracellular FVIII levels.

## 1. Introduction 

Hemophilia A (HA) is a bleeding disorder caused by lack of or a defective Factor VIII (FVIII) protein [1, 2] and results from defects in the *Factor 8* (*F8*) gene. Infusion of plasma-derived or recombinant (r)-FVIII protein permits the efficient management of this chronic disease [3]. The most significant adverse event, which occurs in approximately 20% of patients overall, is the development of antidrug antibodies which inhibit infused FVIII function [4, 5].

The *F8* gene is large gene that contains of 26 exons and spans 186 kb at Xq28 region of X chromosome [6]. An assessment of genetic defects in 1000 hemophilia subjects showed point mutations, inversions, deletions, abnormal splice sites, and so forth, in the *F8* gene [7]. 

In addition to mutations, the lack of expression of the endogenous *F8* gene is also a risk factor for the development of inhibitors [8, 9]. For example, a previous report demonstrated expression of the truncated FVIII protein in HA patients [10]. These truncated or defective FVIII proteins are synthesized in the cytosol of various cells and tissues and may be trapped inside the lysosomal vacuoles. Clinical studies correlating various mutations in the *F8* gene with prevalence of inhibitors are also generally consistent with the premise that the synthesis of an endogenous FVIII polypeptide chain is necessary for inducing central tolerance. A recent systemic review showed the risk of inhibitor development in patient with different types of *F8* gene mutations [11]. 

Current methods estimate plasma levels of endogenous FVIII using techniques such as ELISA; however, some defective proteins may be synthesized but not secreted. Thus it is also important to develop methods for the intracellular detection of FVIII. Such methods would also be useful to understand the distribution of FVIII in different cells and tissues and better understand intracellular trafficking of FVIII. 

*In vivo* protein expression has been studied predominantly by mRNA-based quantitative PCR. Such methods though useful may not be very informative as mRNA expression levels do not necessarily correspond with protein expression levels [12]. Western blotting and ELISA are routinely used and are better suited methods to detect protein expression *in vivo*. However, these techniques are not appropriate to simultaneously study FVIII protein expression levels in distinct cell types.

Flow cytometry is an established high-throughput technique to identify intracellular levels of protein and as such has become an integral tool in immune monitoring and cancer diagnostics [13–15]. Similarly, the cytoplasmic staining and detection of coagulation factor ADAMTS13 protein have been analyzed previously using flow cytometry [16]. The clinical relevance of high-throughput techniques to quantify intracellular levels of FVIII has been illustrated by the recent finding that FVIII mutation can cause HA even though specific activity is not affected. It was demonstrated that the point-mutation N1922S exhibits specific activity which is comparable to that of the wild-type protein; however, poor secretion of the N1922S mutant leads to mild HA [17]. Flow cytometry is well suited to identify and compare cytoplasmic levels of FVIII in both heterologous expression systems as well as in a patient's cell and tissue samples. 

Here, we optimized an ICS assay based on commercially available monoclonal antibodies to study the endogenous and transient expression level of intracellular FVIII protein in mammalian cell lines such as *NIH-3T3*, *HEK-293,* and *CHO*. We applied this method to detect endogenous expression level of intracellular FVIII protein in human PBMCs.

## 2. Materials and Methods

**Figure 1:**
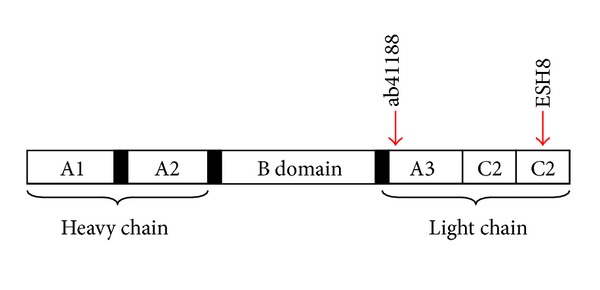
Schematic diagram of FVIII protein showing the binding epitope of anti-FVIII monoclonal antibodies ab41188, and ESH8. The monoclonal antibody, ab41188 binds to the N terminal of A3 domain, while ESH8 binds to the C2 domain of the FVIII protein.

*Reagents.* phosphate buffer saline (PBS, Hyclone) and bovine serum albumin (Acros Organic) were used in preparation of flow cytometry washing buffer. Fixing and permeabilization agents (IntraPrep, Beckman Coulter) were used for ICS. Cells were resuspended in 200 *μ*L of 1% paraformaldehyde (EMS microscopy) in PBS before flow cytometry analysis. Primary mouse anti-human FVIII unlabelled monoclonal antibodies ab41188 (Abcam Inc.) and ESH8 (American Diagnostica Inc.) were used for intracellular staining (Table 1). The monoclonal antibody ab41188 binds to the N terminal of A3 domain of FVIII protein while monoclonal antibody ESH8 [18–20] binds to the C2 domain of FVIII protein. Both antibodies can detect the full-length wild-type protein (Figure 1). Alexa Fluor 488 conjugated goat anti-mouse secondary antibody (Invitrogen) was used to detect the signal from primary monoclonal antibodies used in flow cytometry. 

*Preparation of PBMCs.* heparinized venous blood was collected from unrelated healthy registered blood donors of the NIH Blood Bank. Mononuclear cells were isolated by centrifugation on lymphocyte separation medium (Cellgro, USA). 1 : 1 diluted blood with PBS (pH7.4) was loaded on 5 mL of lymphocyte separation media (LSM-cell grow) in a 50 mL BD Falcon tube. After centrifugation for 30 min at 250 g, buffy coat containing mononuclear cells was collected in a 50 mL tube and washed with PBS twice by centrifugation at 200 g for 10 min to separate the platelets. The viable cell number was obtained using a Cellometer cell counter (Nexcelom Bioscience, MA, USA) following staining with trypan blue. 

*Plasmid Vector.* the full-length recombinant FVIII protein expression vector was used for the transfection studies with various cell lines. The vector was developed to express FVIII protein in a backbone derived from the pcDNA3 plasmid containing an ampicillin resistance cassette. The vector contains cDNA-polyA cassette under the control of a strong CMV promoter and a positive neomycin (Neo) selection marker. The HOW1-WT expression vector contains cDNA corresponding to the H3 haplotype [9] of the human *F8* long isoform. The expression vector was designed by genOway, France and was amplified in *E. coli* and purified using CsCl gradient centrifugation by Loft strand Laboratories (MD, USA). 

*Cell Culture and Transfection.* mouse embryonic fibroblast (*NIH-3T3)*, Chinese hamster ovary (*CHO),* and human embryonic kidney (*HEK-293)* cells (ATCC, VA, USA) [21] were cultured in DMEM supplemented with 10% fetal bovine serum and incubated in 5% CO_2_ at 37°C with slightly modified protocol. Cells in the exponential growth phase were detached with 0.025% trypsin EDTA from a tissue culture dish (Falcon, NJ, USA). 1 × 10^6^ of these cells were inoculated on a 25 cm^2^ flask with 5 mL of DMEM supplemented with 10% FBS. After 24 hours of incubation with 60–80% confluence, the cell growth, fresh complete medium was replenished for an additional 1 hour before transfection. The *F8* constructs were transfected with Gene Jet transfection reagents (Sigmagen Lab, USA) in a DNA Gene Jet (3 : 1) complex ratio. After 6 hours of exposure, transfection reagents containing medium were replaced with complete DMEM culture medium, supplemented with 10% fetal bovine serum (Invitrogen, USA). Cells were harvested 16 hours after transfection and the intracellular FVIII expression was detected by flow cytometry.

*Flow Cytometry Analysis.* transfection efficiency, following the transfection of *NIH-3T3*, *CHO,* and *HEK-293* cells, was estimated by staining with monoclonal antibody to FVIII protein. Cells were harvested and washed twice with washing buffer. Cells were fixed and permeabilized using IntraPrep reagents followed by incubation with diluted (1 : 100) monoclonal antibodies in 0.2% BSA in PBS for 1 hour at RT. The FVIII protein expression was detected by using goat anti-mouse secondary antibody conjugated with Alexa Flour 488. Flow cytometry data was analyzed with FLOWJO (Tree Star, OR, USA). The mean and median fluorescence signal intensity was defined as intensity of cells lying within a predefined gate. The appropriate gating for the live cells was defined on unstained fixed and permeabilized cells on FSC versus SSC dot plot. Same gating parameters were applied for all samples in the same experiment.

## 3. Results

### 3.1. Endogenous Expression of FVIII in Cell Lines

**Figure 2:**
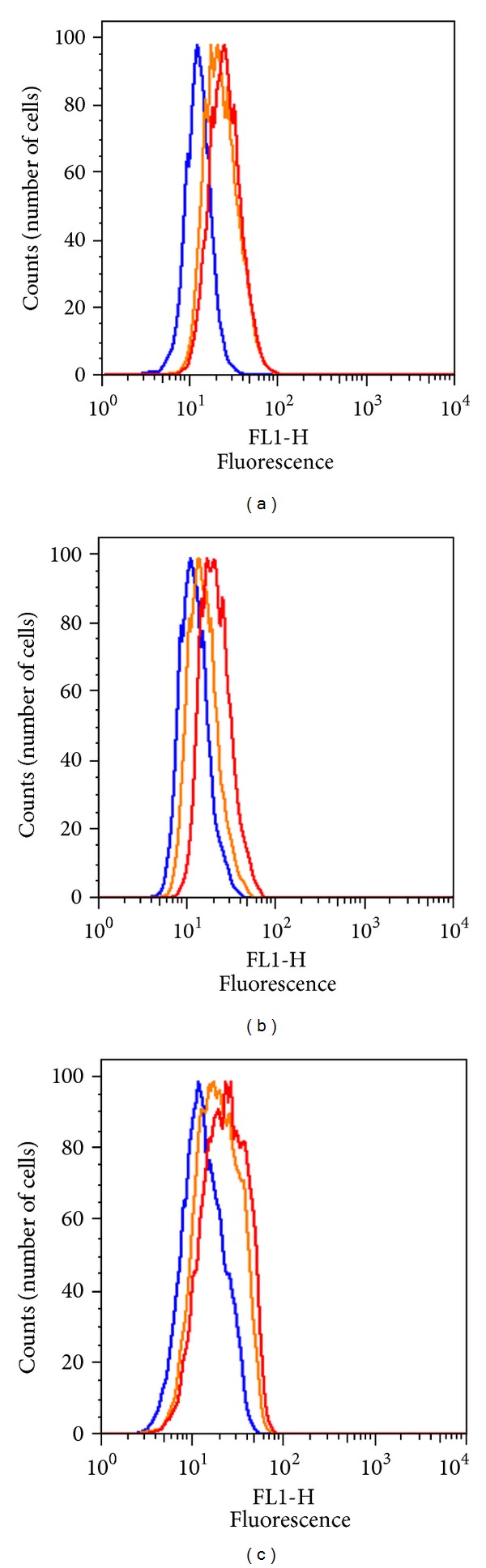
Estimation of endogenous expression of FVIII in untransfected cells. Fixed and permeabilized cells were incubated with anti-FVIII monoclonal antibodies ab41188 and ESH8. Binding of FVIII protein was detected using Alexa Fluor 488 labeled goat anti-mouse IgG secondary antibodies. Each histogram depicts the fluorescence intensity of 10,000 cells stained with isotype control (blue), IgG2a ab41188 (red), and ESH8 (orange). Figures depict the ICS staining in (a) mouse fibroblasts (*NIH-3T3*), (b) Chinese hamster ovary, (*CHO*) and (c) human embryonic kidney (*HEK-293*) cells.

We used a transient expression system for standardizing the flow cytometry assay to detect endogenous FVIII. Several mammalian cell lines have been used to express recombinant proteins. We first determined whether the human anti-FVIII monoclonal antibodies used in the assay detected endogenous FVIII (if any) in three cell lines, *NIH-3T3*, *CHO,* and *HEK-293*. As the antibodies ab41188 and ESH8 are raised against human FVIII, we would expect them to detect FVIII in *HEK-293* cells but not in the rodent cells.  Figure 2 shows an increase in the fluorescence signal compared to the isotype control when all three cell lines stained with the anti-human-FVIII monoclonal antibodies ab41188 and ESH8. This suggests that basal levels of FVIII are expressed in cell lines commonly used for transfection experiments and that anti-human-FVIII antibodies show some cross-species reactivity. 

### 3.2. Expression of Human F8 Gene in Different Cell Lines

**Figure 3:**
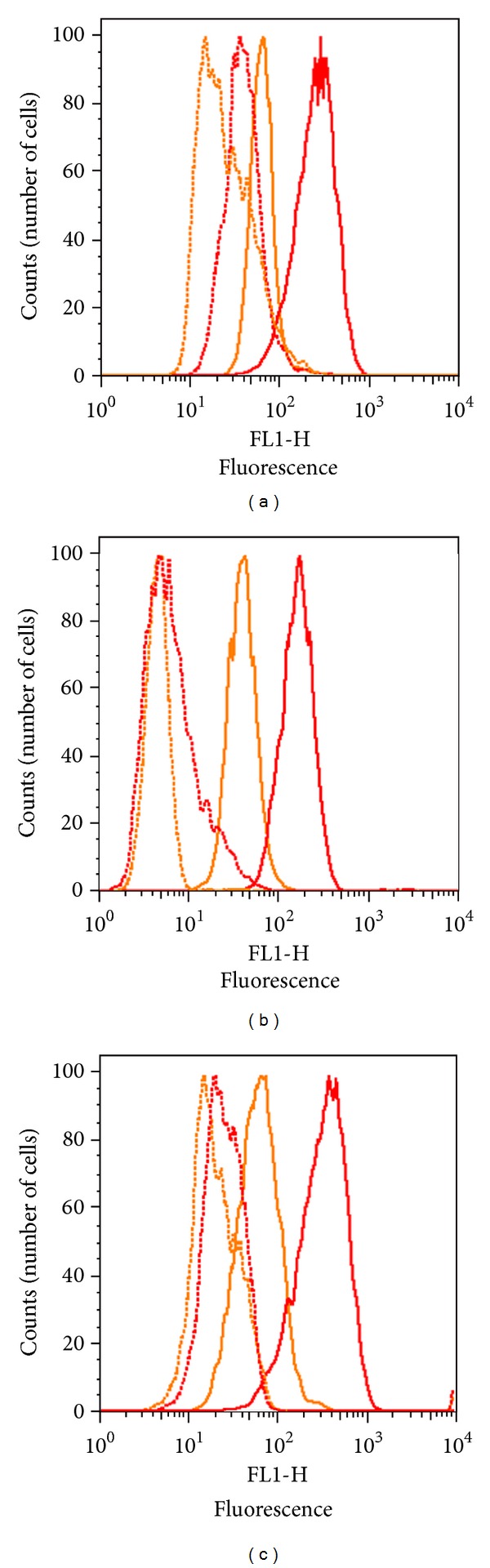
Transient expression of human FVIII in transfected cells. Cells were transfected with plasmid vectors containing wild-type full-length human *F8* gene. 24 hours after transfection, cells were incubated with anti-FVIII monoclonal antibodies ab41188 and ESH8 followed by incubation with Alexa Fluor 488 labeled goat anti-mouse IgG secondary antibodies and detection by flow cytometry. Histograms depict the fluorescence intensity of 10,000 cells stained with ab41188 (red) or ESH8 (orange) antibodies. The untransfected cells are shown with dotted lines and the transfected cells with solid lines. Figures depict the ICS staining in (a) mouse fibroblasts (*NIH-3T3*), (b) Chinese hamster ovary (*CHO*), and (c) human embryonic kidney (*HEK-293*) cells.

We next transiently transfected all three cell lines (*NIH-3T3*, *CHO,* and *HEK-293*) with a wild-type full-length human *F8* plasmid vector. As negative controls we used cells mock-transfected with unrelated vector. After 24 hrs the cells were permeabilized and incubated with anti-FVIII monoclonal antibodies ab41188 and ESH8.  Figure 3 shows increased labeling by both ab41188 and ESH8 in cells transfected with *F8* compared to the untransfected cells. Similarly, cells transfected with *F8* plasmid vector showed a 4–12-fold increase in the fluorescence signal compared to untransfected cells. Thus, increased levels of human FVIII were detected in all three cell lines: *NIH-3T3* (Figure 3(a)), *CHO* (Figure 3(b)), and *HEK-293* (Figure 3(c)) when they were transfected with human *F8* compared to the untransfected controls. 

### 3.3. Expression of Human F8 Gene Is Dose Dependent

**Figure 4:**
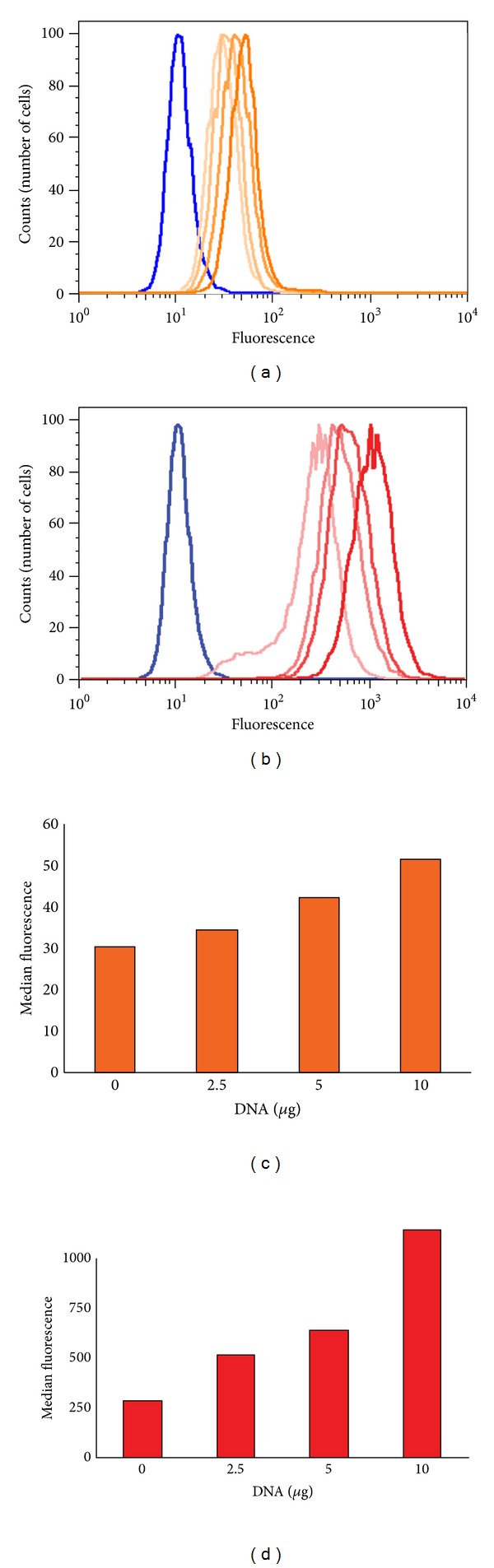
Expression of human FVIII protein is dose dependent. 1 × 10^6^ *NIH*-3*T*3 cells were grown overnight and transfected with 0 *μ*g, 2.5 *μ*g, 5 *μ*g, and 10 *μ*g of full-length human *F8 gene* for 24 hours. Histograms depict the fluorescence intensity of 10,000 cells labeled with ESH8 (a) or ab41188 (b). Cells labeled with the isotype control are depicted in blue, and the intensity of color (light to dark) represents increasing dose of the transfected *F8* vector. The median fluorescence of cells transfected with increasing concentrations of the *F8 *gene is depicted in the lower panels; ESH8 (c) and ab41188 (d).

To further demonstrate that the flow-cytometry assay being developed is specific to FVIII, we investigated the dose dependency of the fluorescent signal. *NIH-3T3* cells were transfected with 0, 2.5, 5, and 10 *μ*g of human *F8* plasmid vector as described above and labeled with the monoclonal antibodies ab41188 and ESH8. The histograms (Figures 4(a) and 4(b)) show that there is an increase in the fluorescent signal with increasing concentrations of *F8* plasmid vector. Moreover a plot of the median fluorescence at each concentration of the *F8* plasmid vector shows a dose-dependent increase in the signal for both antibodies (Figures 4(c) and 4(d)). These data suggest that the assay detects the specific FVIII protein that is being transfected.

### 3.4. Expression of Human F8 Gene in Human PBMCs

**Figure 5:**
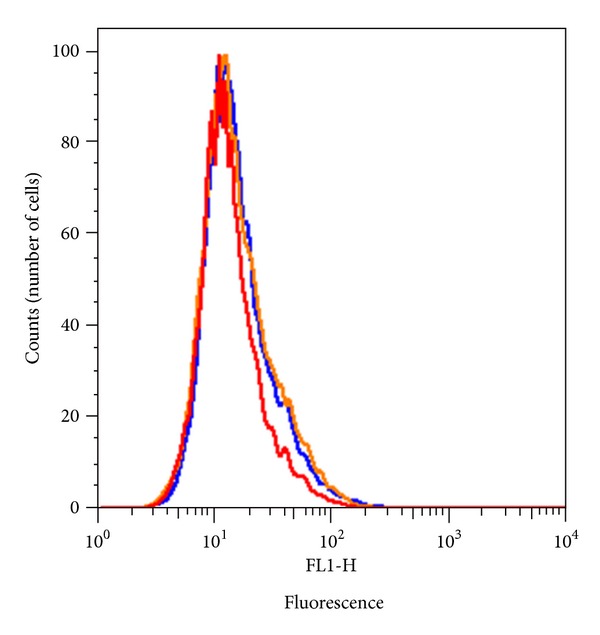
No shift in fluorescence intensity in unpermeabilized PBMCs. Representative histogram showing flow cytometry analysis of human unpermeabilized PBMCs stained with monoclonal antibodies ESH8 (orange) and ab41188 (red). Minimal shift in the fluorescence intensity of the anti-FVIII antibodies was observed compared to the isotype control (blue).

**Figure 6:**
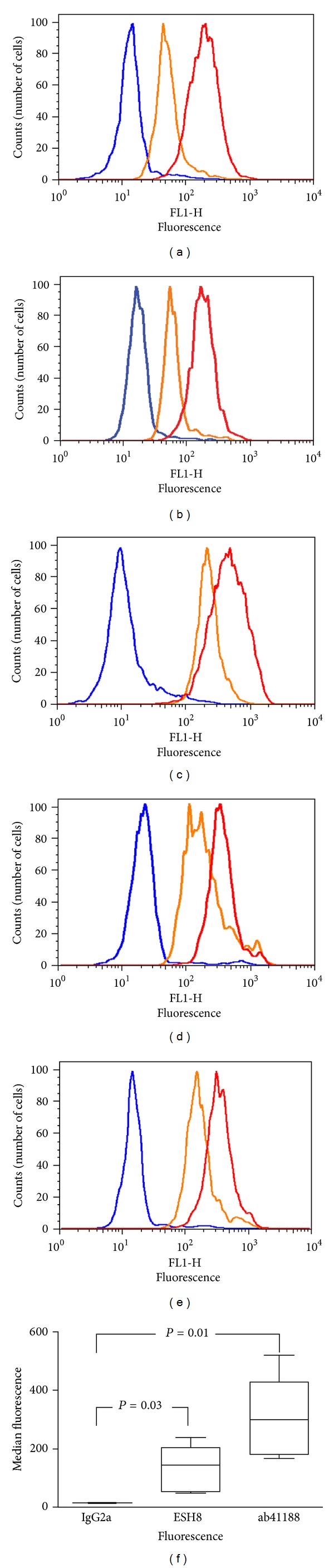
Expression of endogenous FVIII protein in human PBMCs. (a–e) PBMCs were isolated from five unrelated human donors using lymphocyte separation medium and gradient centrifugation. Fixed and permeabilized PBMCs were incubated with anti-FVIII monoclonal antibodies ab41188 and ESH8. Binding of FVIII protein was detected using Alexa Fluor 488 labeled goat anti-mouse IgG secondary antibodies. Each histogram depicts the fluorescence intensity of 10,000 cells labeled with the isotype control IgG2a (blue), ab41188 (red), or ESH8 (orange). (f) Bar and whisker plots show the median fluorescence of all five donors when using IgG2a isotype control antibody and the FVIII-specific antibodies ESH8 and ab41188. On each box, the central mark is the median, the edges of the box are the 25th and 75th percentiles, and the whiskers extend to the most extreme data points. The fluorescence is significantly higher when the cells are labeled with the FVIII-specific antibodies ESH8 and ab41188, than when labeled with the isotype control antibody, IgG2a (*P* = 0.03 and 0.01, resp.).

To demonstrate the utility of this assay in detecting intracellular FVIII in human samples, PBMCs obtained from blood-bank donors were stained with ab41188 and ESH8 antibodies according to the method developed above. Minimal shift in the fluorescence of the anti-FVIII antibodies was observed compared to the isotype control antibodies in nonpermeabilized cells (Figure 5). The flow-cytometry staining analysis with permeabilized PBMCs cells shows that, there is a 10–20-fold increase in fluorescence intensity when anti-FVIII antibodies are used compared to the isotype control antibodies (Figures 6(a), 6(b), 6(c), 6(d), and 6(e)). 

## 4. Discussion

There is an unmet need for a robust assay to detect intracellular FVIII. Currently the extracellular levels of FVIII protein are often estimated to determine the so-called cross-reactive material (CRM) status of hemophilia A patients. Here we have developed a flow-cytometry-based assay to estimate intracellular levels of FVIII. 

We used a transient expression system to standardize the flow-cytometry-based assay. Recombinant human FVIII protein products have been produced in *CHO* cells for many years [22–24]. *NIH-3T3* cells have also been used to express FVIII protein *in vitro* by retroviral vector-mediated F8 gene transfer [25]. Recently, *HEK-293* cells have been used to produce recombinant human FVIII protein in laboratory scale [26]. Therefore, we selected these cell lines for present investigation. 

We demonstrate that while endogenous FVIII can be detected in the cell lines *NIH-3T3*, *CHO,* and *HEK-293,* there is a significant increase in the fluorescence signal when the cells are transfected with human *F8 *(Figures 2 and 3). Although *HEK-293* cells are of human origin and thus the endogenous FVIII should be detected with the human anti-FVIII antibodies used in this study, they showed the lowest levels of protein (Figure 2). This suggests that the expression of endogenous FVIII in *HEK-293* cells is indeed low. 

When an equivalent amount (10 *μ*g) of *F8* plasmid vector was used to transfect all three cells lines, *NIH-3T3* cells showed the highest (3-fold) increase in the fluorescence signal compared to the nontransfected cells. In addition the *NIH-3T3* cells showed higher transfection efficiency with consistent intracellular FVIII expression (Figure 3). We therefore used this expression system to determine the dose dependence of the assay. 

Using *NIH-3T3* cells transfected with *F8* plasmids vector we demonstrate clear dose dependence (Figure 4) in the increase in the fluorescence signal when either ab41188 or ESH8 was used as the detector antibody. This result demonstrates that the detection of FVIII in the flow-cytometry assay is specific and that the assay can be used for semiquantitative measurements. 

The transient expression system coupled with flow cytometry to detect intracellular levels of FVIII-based assay would be very useful in studying the intracellular trafficking or wild-type proteins as well as mutants and polymorphic variants of the protein. Thus, for example, there are over 2500 reports that describe 898 unique missense mutations in hemophilia A patients (for comprehensive data base, see http://hadb.org.uk/). It is not clear whether all these mutations result in a defective FVIII with respect to activity. It is plausible that at least some of these mutations cause the disease due to improper trafficking resulting in the protein not being secreted as has been recently demonstrated for the FVIII mutant, N1922S [17]. 

In an HA patient, on the other hand, the expression (or lack thereof) of FVIII is an important factor in determining the risk of developing inhibitors. A flow-cytometry-based assay to estimate the intracellular levels of FVIII used in conjunction with an ELISA to determine the plasma levels of FVIII in HA patients would be a useful addition. We therefore demonstrated the utility of this assay in detecting FVIII in PBMCs obtained from normal donors (Figure 6). Fluorescence-based assays can show considerable variability day to day. However, the box and whisker plot of the pooled data (Figure 6(f)) clearly show that PBMCs labeled with either of the FVIII-specific antibodies, ESH8 or ab41188, exhibit significantly higher median fluorescence intensity than cells labeled with the isotype control. 

The semiquantitative flow cytometry-based method described here can be used to detect and compare intracellular FVIII levels. As the method can reliably estimate intracellular FVIII levels in PBMCs, it can be used to compare intracellular levels of FVIII in patient samples to correlate genetic defects with hyposecretion of the protein. Identification of patients with *F8* mutations that result in a protein with wild-type specific activity but with impaired secretion may benefit from small-molecule-based treatment strategies aimed at rescuing the trafficking of the protein. In addition, by estimating both the intracellular and plasma levels of FVIII in patient samples, it may be possible to more accurately reevaluate this important risk factor for immunogenicity. 

## 5. Conclusions

We have developed a reliable and robust flow cytometric method to detect intracellular levels of FVIII in a transient expression system as well as in human PBMCs. 

## Figures and Tables

**Table 1:** Antibodies used to detect expression of FVIII by flow cytometry.

Antibodies	1 × 10^6^ cells		
	Isotype	Concentration (mg/mL)	Dilution
Isotype	IgG2a	0.1	1 : 100
ESH8	IgG2a	0.1	1 : 100
ab41188	IgG2a	0.1	1 : 100
